# A review on nanostructured silver as a basic ingredient in medicine: physicochemical parameters and characterization

**DOI:** 10.3762/bjnano.12.36

**Published:** 2021-05-14

**Authors:** Gabriel M Misirli, Kishore Sridharan, Shirley M P Abrantes

**Affiliations:** 1 Physical Chemistry Laboratory, Bio-Manguinhos, Oswaldo Cruz Foundation (FIOCRUZ), Av. Brasil, 4365, Rio de Janeiro, RJ, Brazil; 2 Department of Nanoscience and Technology, School of Chemical and Physical Sciences, University of Calicut, P.O. Thenhipalam 673635, Kerala, India; 3 National Institute for Quality Control in Health, Oswaldo Cruz Foundation (INCQS, FIOCRUZ), Rio de Janeiro, RJ, Brazil

**Keywords:** bactericidal agent, {111} facets, mechanism of action, silver ion, silver nanoparticles, quality control, virucidal agent

## Abstract

Recent studies with silver nanoparticles (AgNPs) and the history of silver metal as a broad-spectrum bactericidal and virucidal agent, places silver as one of the future biocidal candidates in the field of nanomedicine to eliminate bacteria and viruses, especially multidrug resistant ones. In this review, we have described the various morphologies of AgNPs and correlated the enhanced bactericidal activity with their prominent {111} facets. In addition to prioritizing the characterization we have also discussed the importance of quantifying AgNPs and silver ion content (Ag^+^) and their different mechanisms at the chemical, biological, pharmacological, and toxicological levels. The mechanism of action of AgNPs against various bacteria and viruses including the SARS-CoV-2 was analyzed in order to understand its effectiveness as an antimicrobial agent with therapeutic efficacy and low toxicity. Further, there is the need to characterize AgNPs and quantify the content of free Ag^+^ for the implementation of new systematic studies of this promising agent in nanomedicine and in clinical practice.

## Review

### Introduction

Silver is one of the oldest bactericidal agents in history and is also employed as a broad-spectrum antiviral agent. There are records that date back more than 3,500 years BC referring to its medicinal use in prehistoric Egypt, traditional Chinese medicine, and in the Indian Ayurveda (1000 BC). Aristotle, considered as the father of methodical science and inventor of logic, advised Alexander the Great (335 BC) to add silver to his water [1–3]. Since then, the bactericidal effect of silver nanoparticles (AgNPs) has been studied and several experimental evidences have greatly improved the understanding of its mechanism and effects on the human body and on the environment [4–15].

In recent years, the large scale production and usage of various synthetic organic compounds, also known as emerging pollutants, can be potentially toxic to our ecosystem and health even at very low concentrations. These chemicals are used in industries for the manufacture of pharmaceutical formulations, personal hygiene items, and food products. Emerging pollutants, normally found in domestic and industrial effluents, are able to induce bacterial resistance to drugs and genetic exchange [16–17]. At the same time, the resistance of bacteria to antibiotics is dramatically increasing and the World Health Organization (WHO) has marked this concern as one of the greatest threats to health [18]. It is known that viruses are a major cause of illness and death in the world. However, even after much effort, vaccines could not be developed against some viral pathogens, such as the human immunodeficiency virus (HIV), due their high genetic variability which changes rapidly. Viruses have the fascinating ability to adapt to their host, move to a new host, and escape antiviral measures, and similarly, bacteria can also become resistant to antibiotics [19–21]. Estimates suggest that infections, due to an increased resistance of bacteria and viruses to antimicrobial agents, cause at least 700,000 deaths each year. One scenario, developed by the WHO, predicts that this number could increase to 10 million per year by 2050 if no action is taken to limit antimicrobial resistance [22]. Antibacterial, antifouling, and antibiofilm effects of AgNPs have been extensively studied and reveal that they are lethal to bacteria and effectively prevent the formation of biofilms [23]. This suggests that AgNPs can be incorporated into matrices or materials used in the manufacture of medical devices to prevent adhesion, colonization, and formation of microbial biofilms on the surfaces of these devices. Moreover, the history of AgNPs as a broad spectrum microbicidal agent places it as a viable candidate to be one of the basic ingredients of antibiotics to be used in the future against the increase of multidrug-resistant pathogenic strains. In addition, AgNPs can also be used to combat neglected diseases such as dengue, leishmaniasis, malaria, schistosomiasis, and trypanosomiasis (Chagas disease) among other applications [5,19,24].

The unprecedented increase in multidrug-resistant microorganisms in recent years has call the attention of the scientists to the exploitation of silver and silver nanoparticles as antimicrobial agents [25]. Unique properties of AgNPs present a reasonable alternative for the development of new biocides owing to its lethal activity against bacteria and viruses, while toxicological studies indicate its safe usage in the human body [8,26]. In the field of multiresistant microorganisms, it is reported that AgNPs have a considerable bactericidal effect on multiresistant bacteria due its ability to simultaneously penetrate through the biofilm and attack bacteria on different targets [27]. Studies have verified the effectiveness of AgNPs against different pathogens resistant to drugs of clinical importance, including *Pseudomonas aeruginosa*, ampicillin-resistant *Escherichia coli* O157:H7, and erythromycin-resistant *Streptococcus pyogenes* [28]. Due to the wide range of targets that interact with silver in the organisms, it is unlikely that microbes will develop resistance against it. It is also unlikely that microbes simultaneously develop a series of mutations to protect themselves, unlike in cases in which conventional target-specific antibiotics are administered [29]. In addition, studies indicate a synergistic effect of conventional antibiotics (amoxicillin, gentamicin, ampicillin, streptomycin) with AgNPs, which have proven to be highly efficient for the treatment of bacterial infections [30–31]. However, adequate characterization is essential for the research and development of new drugs incorporated with AgNPs and for the advancement of new possibilities for their medicinal use [32].

#### Morphology of AgNPs and their prominent {111} facets

**Figure 1:**
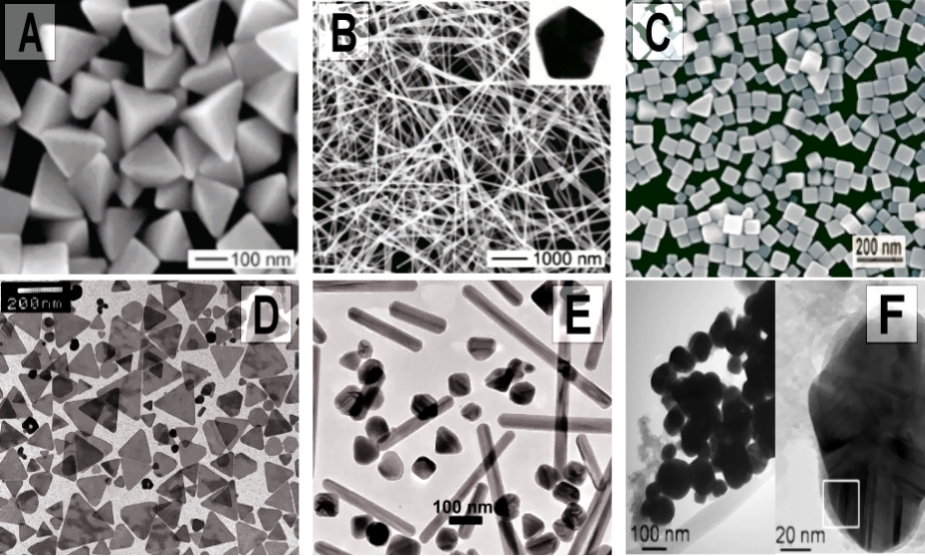
Scanning electron microscope (SEM) with different morphologies: (A) bipyramidal, (B) nanowires, (C) nanocubes and transmission electron microscopy (TEM) micrographs of AgNPs with different morphologies: (D) nanoplates, (E) mixture of spheres and nanorods, (F) spherical bimetallic core–shell Ag@Fe, silver core with iron shell. Part A reprinted with permission from [37], Copyright 2006 American Chemical Society; part B reprinted with permission from [38], Copyright 2005 American Chemical Society B; part C was reprinted from [39], Chemical Physics Letters, vol. 427, by J. M. McLellan, A. Siekkinen, J. Chen, Y. Xia, Comparison of the surface-enhanced Raman scattering on sharp and truncated silver nanocubes, 122–126, Copyright (2006), with permission from Elsevier; part D was reprinted from [40], Advanced Functional Materials, vol. 16, by V. Bastys, I. Pastoriza-Santos, B. Rodríguez-González, R. Vaisnoras, L. M. Liz-Marzán, Formation of Silver Nanoprisms with Surface Plasmons at Communication Wavelengths, 766–773, Copyright (2006), with permission from Elsevier; part E was reprinted from [41], Materials Chemistry and Physics, vol. 114, by J. Jiu, K. Murai, D. Kim, K. Kim, K. Suganuma, Preparation of Ag nanorods with high yield by polyol process, 333–338, Copyright (2009), with permission from Elsevier; part F was reprinted from [42], Optical Materials, vol. 35, by K. Sridharan, T. Endo, S.-G. Cho, J. Kim, T. J. Park, R. Philip, Single step synthesis and optical limiting properties of Ni–Ag and Fe–Ag bimetallic nanoparticles, 860–867, Copyright (2013), with permission from Elsevier.

**Figure 2:**
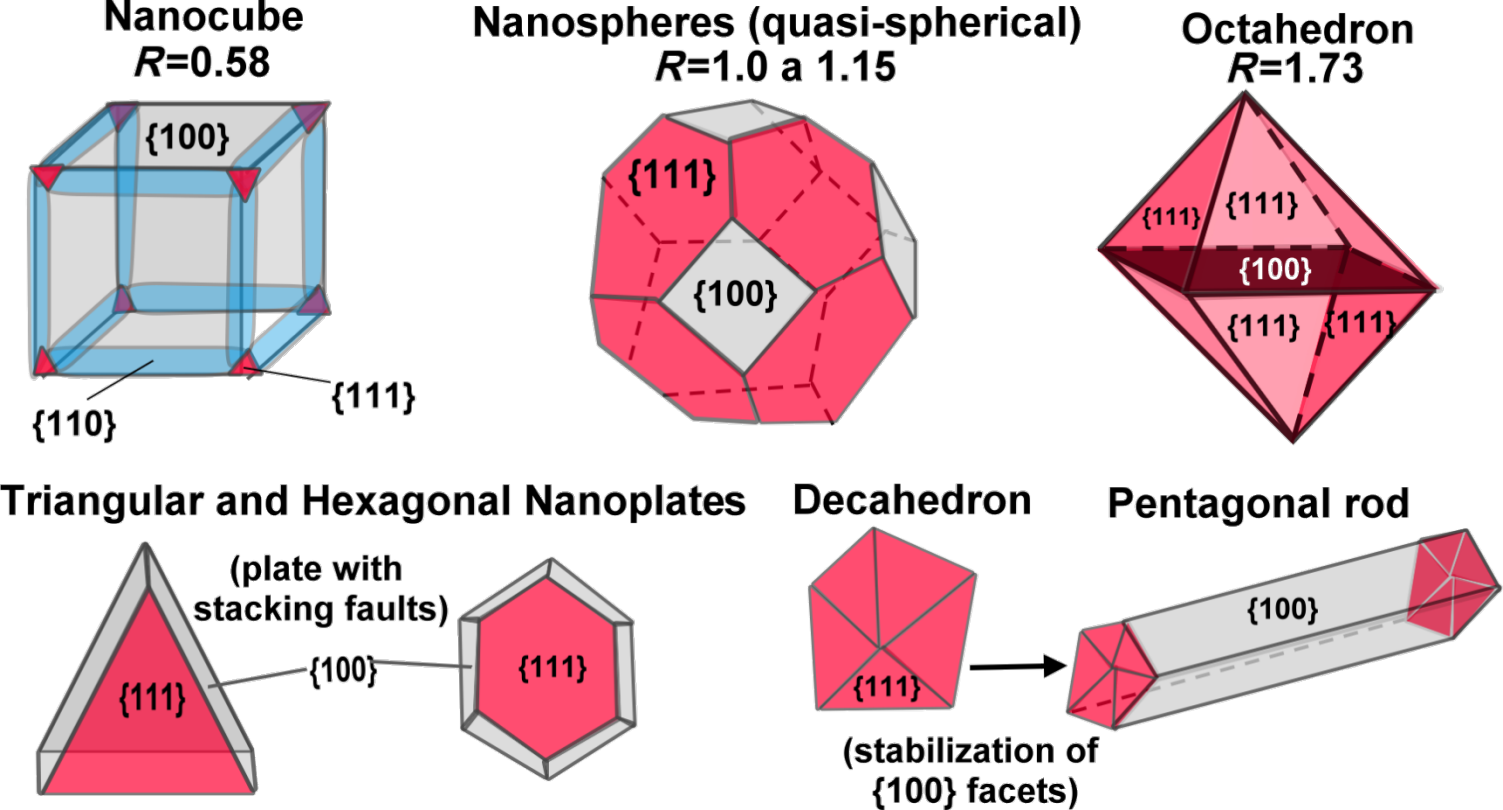
Geometric shapes of nanocrystals with their predominant facets in each structure and the ratio of {111} facets in relation to {100}. Redrawn from [36].

AgNPs can be fabricated with various morphologies, such as spherical, toothpicks, wires, triangular plates, hexagonal plates, pyramids, and cubes depending on the synthesis method used, as depicted in Figure 1. Moreover, they all have specific characteristics, both from the physicochemical and biomedical application points of view [33]. The unique chemical and physical properties of AgNPs are determined not only by the large part of the surface atoms, but also by the crystallographic orientation on their surface. The number of atoms on the surface is influenced by the size of the nanoparticle, while the crystallographic orientation depends on the shape of the particles. The surfaces with {111}, {100}, and possibly {110} exposed facets in AgNPs are different not only in the densities of the surface atoms, but also in their electronic structure, bonding, and chemical reactivity [34–36]. Figure 2 depicts these surfaces (facets) more clearly.

Agnihotri et al. studied the bactericidal activity against *E. coli*, *Bacillus subtilis*, and *Staphylococcus aureus* of spherical AgNPs of various sizes and concluded that their effectiveness increased with decreasing size, regardless of the bacterial strains [43]. On the other hand, studies indicate that the reactivity and antibacterial activity of AgNPs are stronger in particles with predominant {111} facets with high atomic density [29,44]. Thus, truncated triangular nanoplates have greater bactericidal power compared to other morphological forms of AgNPs which contain fewer {111} facets, such as nanospheres, nanorods, and nanocubes. Pal et al. and Dong et al. compared the bactericidal activity of AgNPs with spherical and nanoplate-like morphology and concluded that the nanoplates had a greater biocidal activity due to the {111} facets when compared with nanospheres [29,45]. Acharya et al. evaluated the efficiency of spherical and rod-shaped AgNPs regarding their antibacterial activity against various Gram-positive and Gram-negative bacteria. Death kinetics confirmed that the mortality rate of *Klebsiella pneumoniae* was higher when exposed to spherical AgNPs with larger {111} facets in comparison to rod-shaped AgNPs. Therefore, the {111} facets were found to be responsible for the antibacterial activity of AgNPs, a fact that was confirmed by a death study whose kinetics were supported by the arrangement of Ag-resistant genes in the genome of the test organism [46].

Conversely, AgNPs with different sizes exhibit different antimicrobial activities: smaller particles are more efficient to kill bacteria than larger ones. This could be attributed to the higher surface areas that facilitate the attachment to cell membranes [8]. In addition to the different sizes and shapes that AgNPs can have, other metallic layers can be added to AgNPs, adding new features to the nanoparticles. For example, with the addition of nickel or iron in the production of bimetallic silver nanoparticles, Ag@Ni or Ag@Fe, respectively [42], the nanoparticles acquire magnetic properties. These magnetic nanoparticles have the potential to be used in biomedical applications, such as in therapies that involve magnetic manipulation with photothermal effect promoting a localized bactericidal activity [42,47–49].

#### Properties and oxidative dissolution

The oxidative dissolution of AgNPs occurs by the oxidation of silver to silver oxide (Ag_2_O), with release of Ag^+^ in solution and exposure of unoxidized metallic silver, which may undergo additional oxidation leading to the corrosion of AgNPs [50]. This oxidative dissolution can be influenced by extrinsic factors, such as artificial/solar light and storage temperature as well as by intrinsic factors, such as pH, dissolved oxygen, and zeta potential (ZP) [51–53]. The storage temperature is a critical factor that controls the percentage of oxidative dissolution of AgNPs. The higher the temperature, the greater the speed of the dissolution reaction [54–55]. Kittler et al. studied the dissolution of 50 nm AgNPs dispersed in water at various temperatures and observed that the release of Ag^+^ was ≈5% at 5 °C, ≈50% at 25 °C, and ≈90% at 37 °C. The oxidative dissolution of AgNPs involves reactions with protons (H^+^) and dissolved O_2_. The dissolution of AgNPs is favorable under an acidic pH and can be described by a first-order kinetic reaction, according to Equation 1 [56]. Experimental studies have shown that the decrease in pH generally increases the dissolution kinetics [51,55,57].

[1] $$2\text{Ag}+\frac{1}{2}{\text{O}}_{2}+{\text{2H}}^{\text{+}}\underset{}{\to }{\text{2Ag}}_{}^{+}+{\text{H}}_{2}{\text{O}}_{}$$

**Figure 3:**
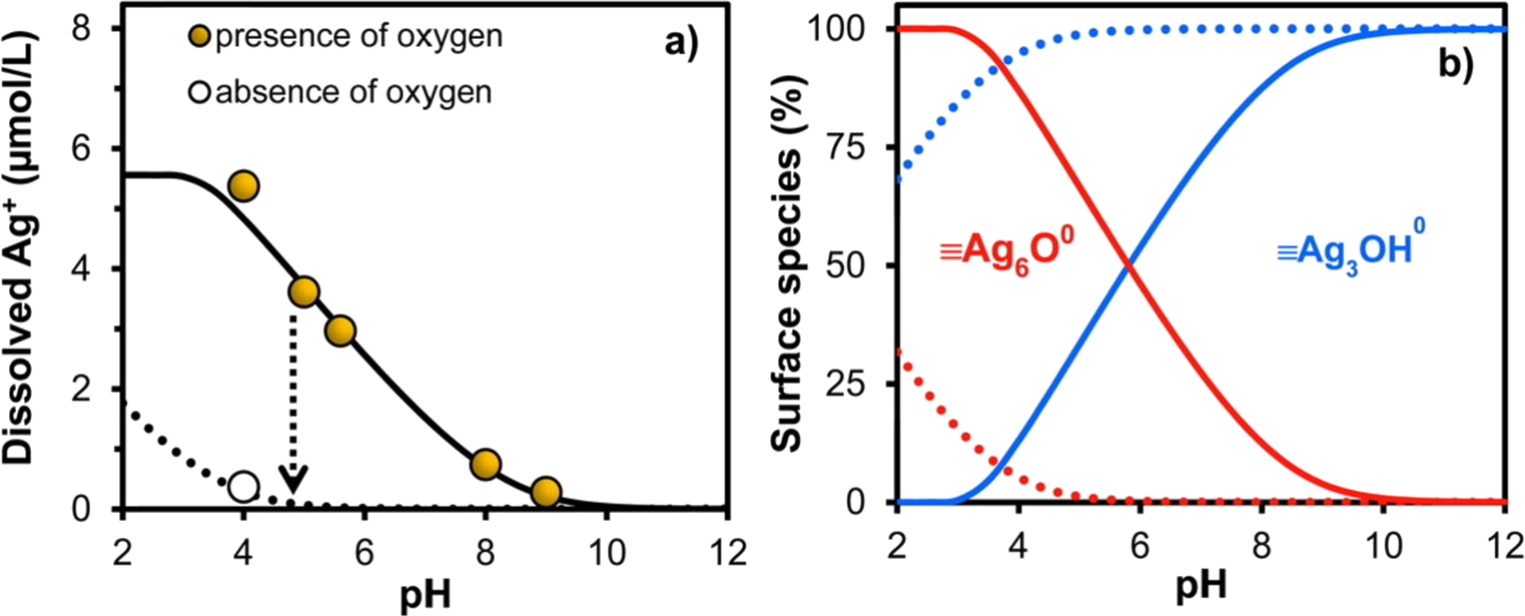
(a) Ag^+^ release after one day of reaction in the presence of oxygen. The dotted arrow indicates decreased Ag^+^ release when AgNPs is Ar-purged. In this case, there is no dissolved oxygen available for oxidative release. (b) Speciation curve for the formation of Ag_6_O^0^ and Ag_3_OH^0^ species as a function of pH. The dotted lines refer to a system without oxygen. Reprinted with permission from [57], Copyright 2015 American Chemical Society.

Liu and Hurt studied the dissolution of AgNPs in buffered solutions (acetate and borate buffer) in a pH range from 4.0 to 9.0, and observed that by increasing the pH from 4.0 to 9.0 it resulted in 10 times less release of Ag^+^. The formation of Ag_3_OH^0^ species as a protective layer was noted while the AgNPs were treated above pH 9.0. Their increased stability was attributed to the lower concentration of dissolved oxygen and due to Ag_3_OH^0^ species acting as a shield and hindering the dissolution process of the AgNPs, as detailed in Figure 3 [55–57].

#### Stability

**Figure 4:**
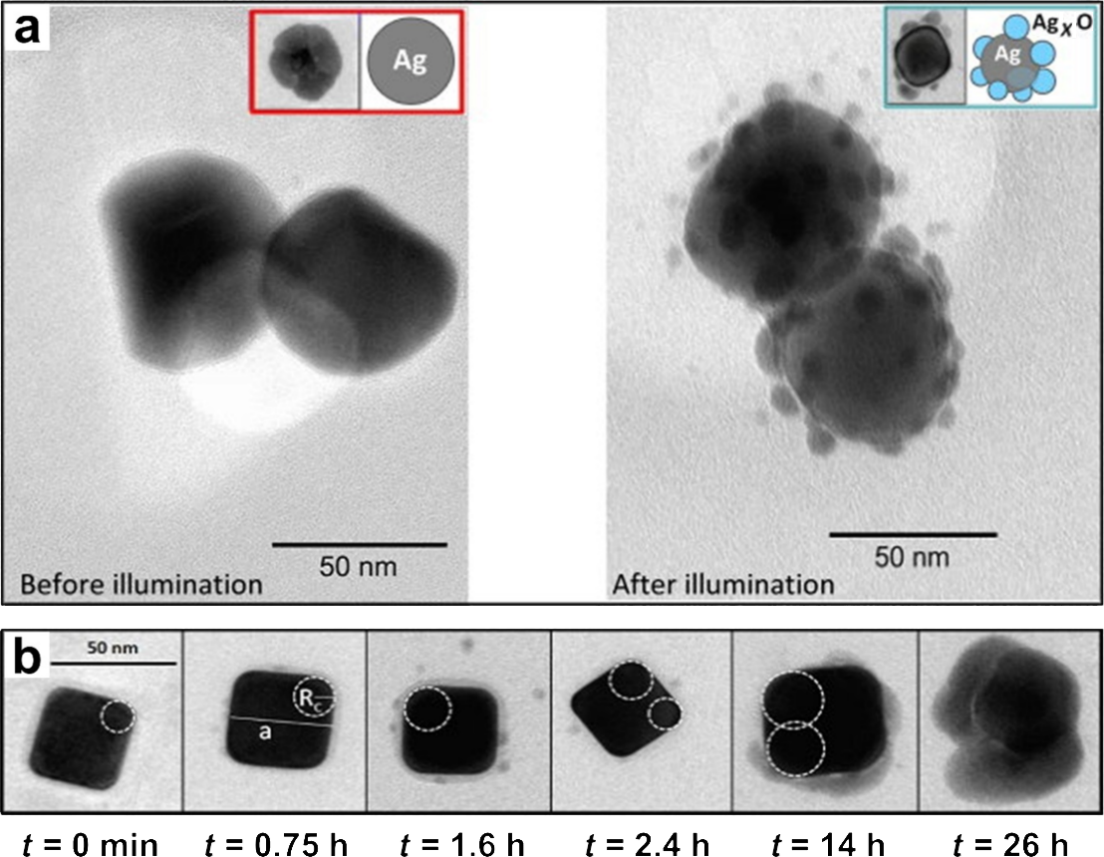
(a) Comparative TEM micrographs of spherical AgNPs before (left) and after (right) light irradiation. The formation of small NPs (Ag*_x_*O) after light irradiation is indicative of photo-oxidation. (b) TEM micrographs representing the cubic AgNPs that were continuously irradiated with light for *t* = 0 min, 45 min, 1.6 h, 2.4 h, 14 h, and 26 h, respectively. Reprinted with permission from [52], Copyright 2013 American Chemical Society.

Another factor that considerably changes the stability of AgNPs is the exposure to artificial or natural sunlight [53]. Grillet et al. studied the effect of light irradiation on AgNPs with spherical and cubic morphologies under ambient conditions. They confirmed that light wavelengths below 495 nm, corresponding to the UV/blue part of the electromagnetic spectrum, were mainly responsible for their photo-transformation [52]. In both the cases, the effect of photoaging was studied by evaluating the impact of the incidence of artificial light (tungsten filament, color temperature of 3200 K) with a power of 1 μW focused on an area of 1 μm^2^. The formation of a Ag*_x_*O shell layer (i.e., the oxidation volume) was theoretically predicted and also experimentally analyzed by UV–vis extinction spectra and transmission electron microscopy (TEM). The rate of photo-oxidation in spherical AgNPs was homogeneous and surprisingly inhomogeneous in the case of cubic AgNPs, which was attributed to the difference in morphology. The noticeable formation of small NPs (Ag*_x_*O) around the spherical AgNPs after light exposure, observed in the TEM micrograph shown in Figure 4a, confirmed the quicker photo-oxidation process [52,58]. After 40 h of light irradiation, the level of oxidation in cubic AgNPs was about 30%, while it took only 9 h of light irradiation for the complete oxidation of spherical AgNPs, confirming that the oxidation process was much slower in the case of cubic AgNPs, as observed from the representative TEM micrographs depicted in Figure 4b.

For a correct assessment, including all studies involving AgNPs at the chemical, pharmacological, biological, and toxicological levels, the identification and quantification of AgNP species as well as the silver ion (Ag^+^) content should be considered, as their behaviors and mechanisms are distinct [59–60]. Although the Ag^+^ ion has antimicrobial properties (such as AgNPs), it is rated as one of the main agents responsible for the toxicity of silver and argyria in our body. When Ag^+^ enters the bloodstream, it is transported bound to albumin and thiol groups. When it reaches a region close to the skin, in areas affected by light, it can easily be photoreduced to AgNPs, which are then immobilized in the epidermis. Immobilization is both physical, due to the low diffusivity of the particles, and chemical, since thiol-exchange reactions occur with Ag^+^ but not with AgNPs. This fact explains why AgNPs are not responsible for causing argyria or argyrosis [1,11].

**Figure 5:**
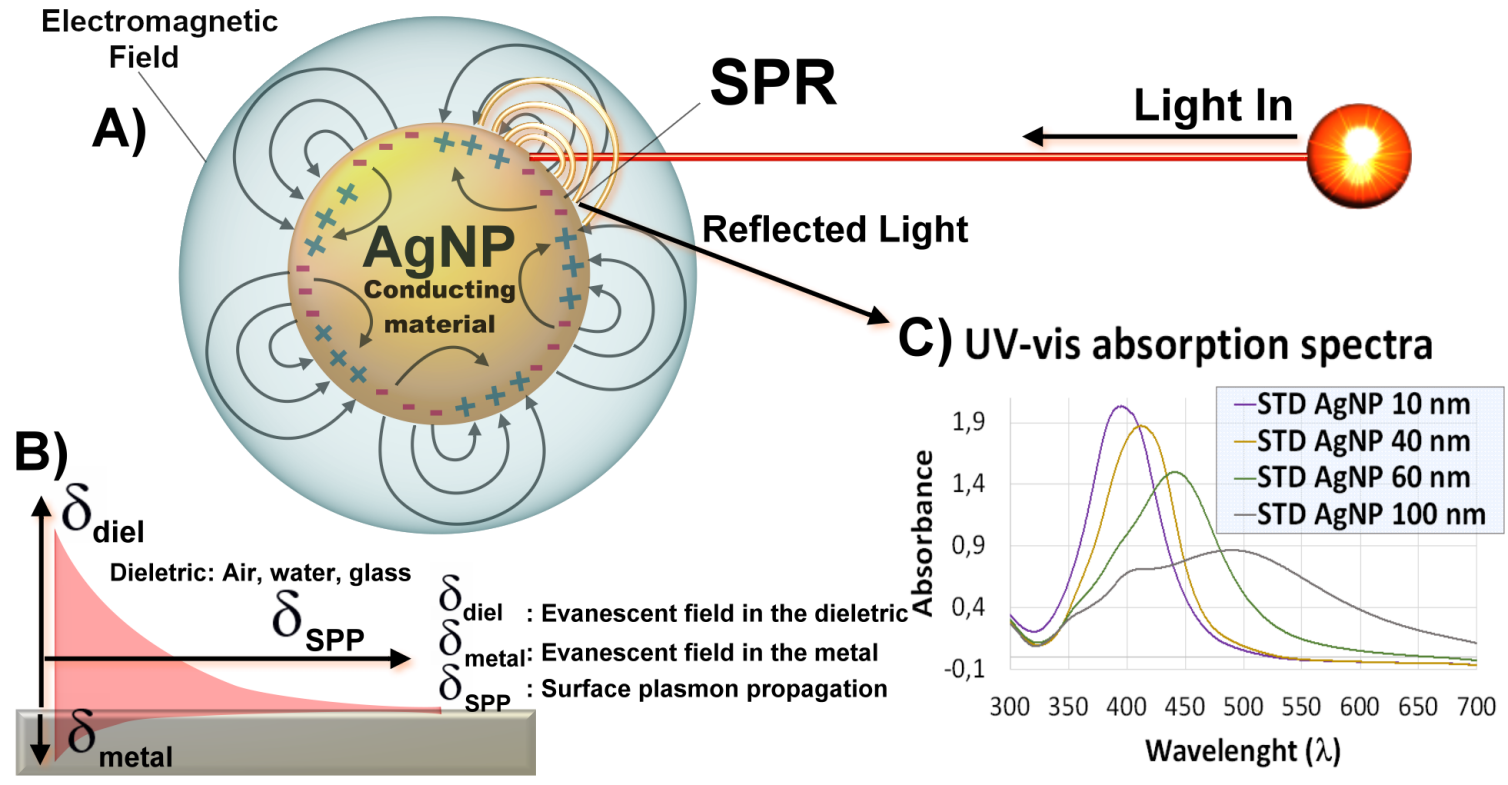
(A) Propagation of surface plasmon resonance in spherical AgNPs during their interaction with the electromagnetic radiation. (B) The order of magnitude of the plasmon lengths in SPR. (C) UV–vis absorption spectra of spherical silver nanoparticle standards with sizes of 10, 40, 60, and 100 nm, respectively, all with a concentration of 0.02 mg/mL.

It is well known that the strong interaction of AgNPs with light leads to surface plasmon resonance (SPR) effect when the incident light frequency coincides with the frequency of the oscillating electrons on the surface. The surface of AgNPs stores the conducting electrons inside the particles and establishes a restorative force which creates a dipolar surface plasmon frequency [61–62]. SPR can be modulated by controlling the shape of AgNPs, which in turn controls the ways in which the electrons oscillate. The light is spread and absorbed inside the nanostructure and intensifies the local electric fields [63]. In general, smaller nanoparticles induce the production of hot electrons from plasmonic metals, while larger size nanoparticles favor the scattering of the light. There is a strong electromagnetic field that is produced by fast and coherent oscillating electrons which extend into the metal and the surrounding environment [62–63]. The term "plasmon" was introduced by Pines and Bohm (1952) and it means, in a very brief way, that electrons move as a group inside of a metal. Together, the electrons alternately flow back and forth while being attracted to the positive ions, which make up the crystal structure, and are repelled when they are very close to each other. In this regard, the electrons could be described as waves, as depicted in Figure 5 [64]. The type of metal, size, shape, the molecules attached to the surface, and the degree of aggregation of the particles determine the energy range (frequency) of the light that can excite the plasmons (Mie scattering theory). For example, the main absorption wavelength (dipole) of spherical AgNPs (10 nm of size) is at ≈400 nm. Larger AgNPs and AgNPs with different shapes, on the other hand, absorb different light wavelengths due to other absorption modes, generally higher than 400 nm, as depicted in Figure 5C [65–66].

#### Biological applications of AgNPs

Due to their unique properties, AgNPs have been widely used in household utensils, in food storage, and in various biological and biomedical applications [33]. Several studies have demonstrated the antimicrobial power of AgNPs against a wide range of bacteria (i.e., Gram-negative [67], Gram-positive [31], and multiresistant bacteria (MR) [27,68–71]), fungi [69,72–74], and viruses [8,75–77] in addition to anti-inflammatory [78–80], anti-cancer [27,81], and anti-angiogenic properties [33,47,82]. Silver has been widely used to heal ulcerative wounds and to treat burns. Due to the antimicrobial and anti-inflammatory properties of AgNPs, these particles can prevent bacterial infection at the wound area and accelerate healing [27,83]. AgNPs have been shown to improve the efficacy of cancer treatments, increasing the effectiveness of drug administration and producing antitumor effects [27,81]. In addition, AgNPs have a central role in the development of new treatments for neglected diseases, which are caused by infectious agents or parasites and are considered endemic in populations at low-income regions. Little is invested in the research and development of new drugs by the pharmaceutical industry to treat these neglected diseases as it is not commercially interesting [84–85]. AgNPs have appeared as a possible alternative in the therapy against many of these diseases as they present low toxicity and improved efficacy [86]. In vivo and in vitro studies have demonstrated the efficacy of AgNPs in treating neglected diseases, such as dengue [87–88], leishmaniasis [89–92], malaria [93–95], schistosomiasis [96–97], and trypanosomiasis (Chagas disease) [98–99].

#### Mechanism of action of AgNPs

One of the most important characteristics of NPs is their high surface-to-volume ratio. Typical in bulk materials, the number of atoms in a volumetric particle found on the surface is insignificant. The opposite occurs in nanometer-sized particles. For instance, a cube with 1 cm of size has about 0.00006% of the atoms on the surface, while a cube with 10 nm of size has approximately 60% of the atoms on the surface. Therefore, many of the physical properties of AgNPs, such as their effectiveness against bacteria, viruses, and biofilm permeability are related to the size of the nanoparticles [7–8]. The atoms on the surface together with the influence of size and shape result in NPs exhibiting behaviors markedly different from the bulk [34,100]. AgNPs have been reported to show much higher bactericidal activity under oxygenated conditions when compared to anaerobic conditions. This suggests that the antibacterial action is in fact due to Ag^+^ ions, which are gradually released during the oxidative dissolution of AgNPs [101]. Furthermore, published studies regarding the bactericidal activity of AgNPs have shown that AgNPs kill bacteria at a very low concentration and, therefore, do not cause acute toxic effects on human cells [102–103].

**Effect on bacteria:** Agnihotri et al. identified the bacteriostatic/bactericidal effect of AgNPs and determined the minimum inhibitory concentration (MIC) and the minimum bactericidal concentration (MBC) of spherical silver nanoparticles against four bacterial strains. For AgNPs smaller than 10 nm, an improved bactericidal activity was evidenced as revealed by the delay in bacterial kinetic growth. Smaller sized AgNPs (5 nm) showed the best results wherein the bactericidal activity was faster against all tested strains when compared to larger sized AgNPs [43]. Regarding Ag^+^ and AgNPs species, there are some differences in the way they act on bacteria. Table 1 shows the main studies and conclusions from each author for a better understanding on this topic.

**Table 1:** Effects of silver ion (Ag^+^) and AgNPs on bacterial cells.

effects	Ag^+^	AgNPs	comments	ref.

block respiratory enzyme and electron transfer	yes	yes	—	[8,12]
interact with DNA	yes	yes	—	[8]
interact with iron–sulfur groups	no	yes	—	[11]
interact with thiol groups in proteins	yes	no	—	[11]
induce the production of ROS (O_2_^−^, H_2_O_2_, OH.)	yes	yes	occurs only if free intracellular iron is present.	[11–12]
form a low molecular weight region at the center of the bacteria.	yes	no	—	[8]
electrostatic charge promotes greater interaction	no	yes	—	[8]
pass through biofilms	yes	yes	AgNPs act mainly in the range from 1 to 10 nm attached to the cell membrane surface.	[8,11]
induce the Fenton reaction and consequently kills apoptotic cells	yes	yes	subsequent oxidation of iron by H_2_O_2_ generates a hydroxyl radical, a powerful oxidant that attacks adjacent DNA.	[11,72,104–105]
“zombie effect”	yes	yes	after triggering apoptosis, AgNPs and Ag^+^ interact with the cellular components of the dead bacteria (RNA, polysaccharides, phospholipids, proteins, and DNA) creating new silver nanoparticles capped by the genetic material of the bacteria (AgNPs–bac).	[106]

**Figure 6:**
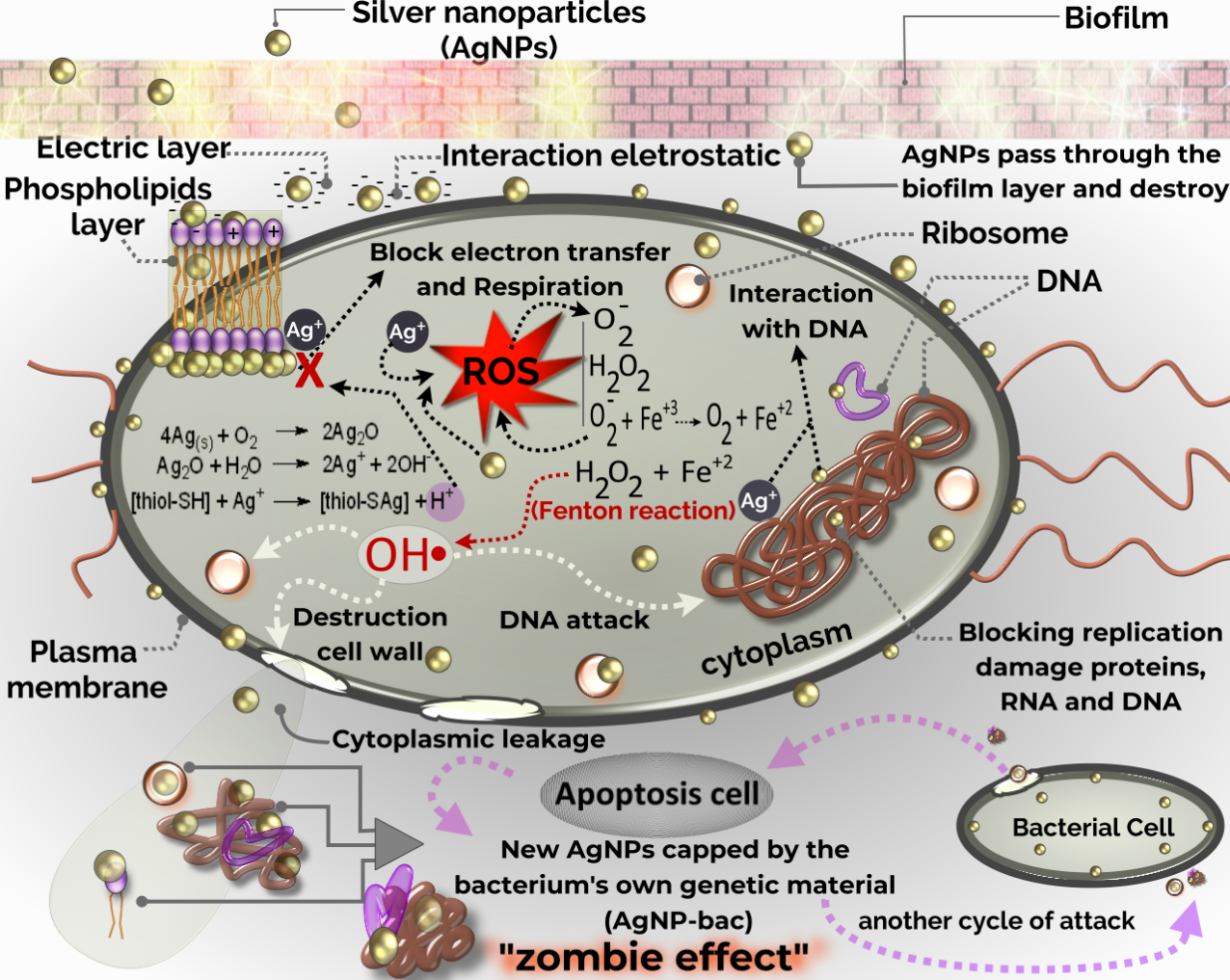
Mechanism of action of AgNPs in the bacterial cell.

AgNPs have not been shown to cause bacterial resistance since, unlike antibiotics, AgNPs do not exert their antibacterial effects in a single specific location, but rather at several levels (e.g., in the bacterial wall and by blocking electron transfer, in cell respiration and replication due to the damage to the proteins, RNA, and DNA [8,107]). In addition, there is substantial evidence that AgNPs produce reactive oxygen species (ROS). The accumulation of intracellular ROS is well known as an important regulator of apoptosis [72]. The production of oxidative species may be due to trapped electrons in the respiratory chain. Antioxidant enzymes are unlikely to detoxify species generated from the damaged respiratory chain since they depend on thiol groups which are occupied by silver ions. The increase in superoxide and hydrogen peroxide anions in the reaction with iron (Fenton reaction), according to Equation 2 [104] and as described in Figure 6, are indicative of the deleterious oxidative changes in the internal structure of cellular proteins, RNA, and DNA leading to redox changes, which in extreme conditions can lead to cell death by apoptosis [11,23,108].

[2]
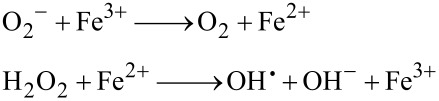


Wakshlak et al. presented a new action mechanism of silver, called the "zombie effect". The AgNPs interact with the cellular components of the dead bacteria (i.e., RNA, polysaccharides, phospholipids, proteins, and DNA,) and are stabilized and capped by the genetic material of the bacteria (AgNPs–bac). According to the Le Chatelier’s principle, AgNPs are redirected to live bacteria with a higher potential for lethality according to Figure 6 [106].

**Effect on viruses:** The emergence of resistant viral strains and the lack of effective antiviral therapies increase the need to develop safe and potent alternatives to conventional antiviral drugs. Metals can attack a wide range of targets in the virus; therefore, there is great interest in studying possible binding mechanisms of NPs to the viral capsid to inhibit further fusion. Furthermore, there is less chance for resistance development against NPs when compared to conventional antivirals [75]. The main antiviral mechanism of AgNPs is probably the physical inhibition of the binding between the virus and the host cell [109]. A viral infection begins with the adhesion or binding of the virus to the host receptors, followed by penetration, replication, and budding. The main antiviral strategies are to effectively block the binding of the virus to the host receptors (i.e, the prophylactic effect) and to inhibit viral replication and budding (i.e, the therapeutic effect) [75–76,110]. In vitro studies reveal that AgNPs can act as inhibitors of viral entry by blocking viral attachment and penetration into cells [109,111–112]. They also inhibit the synthesis of viral negative-stranded RNA and viral budding [76,113]. One of the most protective innate defense mechanisms of the host against viruses is related to the intracellular actions of proteins encoded by interferon-stimulated genes (ISGs), such as interferon-induced proteins with tetratricopeptide repeats (IFITs). IFITs inhibit virus replication by binding to and regulating cellular and viral proteins and RNAs [114]. Studies indicate that AgNPs did not affect cellular viability, according to mitochondrial cytotoxicity tests, or plasma membrane integrity. Interestingly, they exhibited potent ability to activate macrophages to produce activated ISGs and pro-inflammatory cytokines, such as interleukins (IL-6, IL-8) [76,115–117].

Several studies have shown that AgNPs can act against various types of viruses, viz. human immunodeficiency virus type 1 (HIV-1) [111,118], monkeypox virus (MPV) [112], herpes simplex virus type 1 (HSV-1) [118], porcine epidemic diarrhea virus (PEDV, from the coronavirus family) [76], Tacaribe virus (TCRV) [113], and against respiratory pathogens such as adenovirus, parainfluenza, and influenza viruses (H1N1, H3N2) [117,119]. Furthermore, in vivo studies revealed the antiviral ability of AgNPs against infection caused by the respiratory syncytial virus (RSV). Morris et al., by using in vitro and in vivo models in which mice were infected experimentally, concluded that AgNPs effectively reduced RSV replication and pro-inflammatory cytokine production in epithelial cell lines and inside mice lungs [117].

The dependence of the size of AgNPs on their antiviral activity is a crucial factor. Generally, smaller AgNPs have a stronger antiviral activity and the maximum ideal size for studies is 10 nm [8]. However, several theories are still in discussion and may vary according to each viral species and types of AgNPs evaluated. Table 2 shows the effects of some AgNPs for a better understanding of their possible antiviral mechanisms.

**Table 2:** Effects of silver ion (Ag^+^) and AgNPs on viruses and host cells.

effect	n^º^	was Ag+ content in the silver solution reported?	AgNPs (structure, size, concentration)	virus/experiment	ref.

glycoprotein button lock	1	no	AgNPs capped with bovine serum albumin (BSA) and poly-vinylpyrrolidone (PVP). Shape: The distribution of shapes in the sample is broad and a significant amount of nanoparticles are not spherical (e.g., multi-twinned NPs). Size: 6.53 ± 2.41 nm (AgNPs–PVP) and 2.08 ± 0.42 nm (AgNPs–BSA). Concentration: 25 µg/mL.	HIV-1 in vitro	[111]
	2	no	AgNPs capped with mercaptoethane sulfonate (AgNPs–MES). Shape: moderately polydispersed, spherical nanoparticles. Size: 4 ± 1 nm, Concentration: 400 µg/mL.	HSV-1 in vitro	[118]

inhibition and blocking of viruses binding to host cells	3	likewise number 1		HIV-1	[111]
	4	no	silver nanoparticle with chitosan (ch). Shape: spherical. Size: 3.5, 6.5, and 12.9 nm. Concentration: 62–77 µg/mL.	H1N1 in vitro	[109]
	5	no	AgNPs coated with polysaccharide. Shape: not informed Size: 10–80 nm. Concentration: 12.5 µg/mL (size: 10 nm).	MPV in vitro	[112]
	6	no	AgNPs coated with PVP. Shape: spherical. Size: 8–12 nm. Concentration: 50 µg/mL, with the strongest antiviral effect and are not toxic to epithelial cells.	RSV in vitro in vivo	[117]

inhibition of viral RNA synthesis and viral budding	7	yes	Ag_2_S NPs capped with glutathione (GSH). Shape: spherical. Size: 3.7 nm. Concentration: 46 µg/mL.	PEDV (coronavirus family) in vitro	[76]
	8	no	AgNPs uncoated and polysaccharide-coated AgNPs (PS–Ag). Shape: spherical. Size: 10 and 25 nm. Concentration: 25 or 50 μg/mL	TCRV in vitro	[113]

stimulation of the immune system and expression of pro-inflammatory cytokines	9	likewise number 7		PEDV (coronavirus family) in vitro	[76]
	10	likewise number 6		RSV in vitro in vivo	[117]

induction of competition for the virus to bind to the cellular heparan sulfate (HS)	11	likewise number 2		HSV-1 in vitro	[118]

prevention of the virus from coating the endosome	12	likewise number 8		TCRV in vitro	[113]

Respiratory diseases have been increasingly common in recent years, ranging from severe acute respiratory syndrome (SARS) to the most recent and ongoing COVID-19 pandemic caused by the SARS-CoV-2 coronavirus [120]. In this context, the antiviral properties of AgNPs against PEDV [76], H1N1 [109], H3N2 [119], and RSV [117] have already been extensively tested in vitro and in vivo. Recently it has been found that these antiviral properties are also effective in inhibiting SARS-CoV-2 by using AgNPs to coat facial masks [77,120]. These results are suggestive of the development of new measures in which AgNPs can be used to prevent SARS-CoV-2 infection.

#### Biological interactions of AgNPs

When in contact with human plasma, AgNPs adsorb biomolecules such as human serum albumin (HSA), fibrinogen and immunoglobulin (IgG), metallothionein (MT), and ceruloplasmin (CP), forming a protein corona (PC) during silver homeostasis [121–122]. The PC is a highly dynamic system and its composition dynamically changes over time, undergoing various transformations until PC reaches a steady state of constant and stable composition [59,123]. The mechanisms through which PC can influence the absorption of AgNPs from the outside and inside of the cells and its impacts on cellular interactions are still not clear. More research is needed to understand how PC can influence biological and cytotoxic responses [124–125].

Pharmacokinetic and biodistribution data of AgNPs are important for future safe and effective biomedical applications [27]. Liu et al. experimentally verified the interaction between AgNPs (20 nm) and two metalloproteins, metallothionein (MT) and ceruloplasmin (CP), both involved in metal homeostasis. They concluded that protein diversity stabilizes and controls the dissolution and transport of AgNPs. Understanding the interaction between AgNPs and corona proteins is essential to establish new in vivo studies with AgNPs [122].

**Figure 7:**
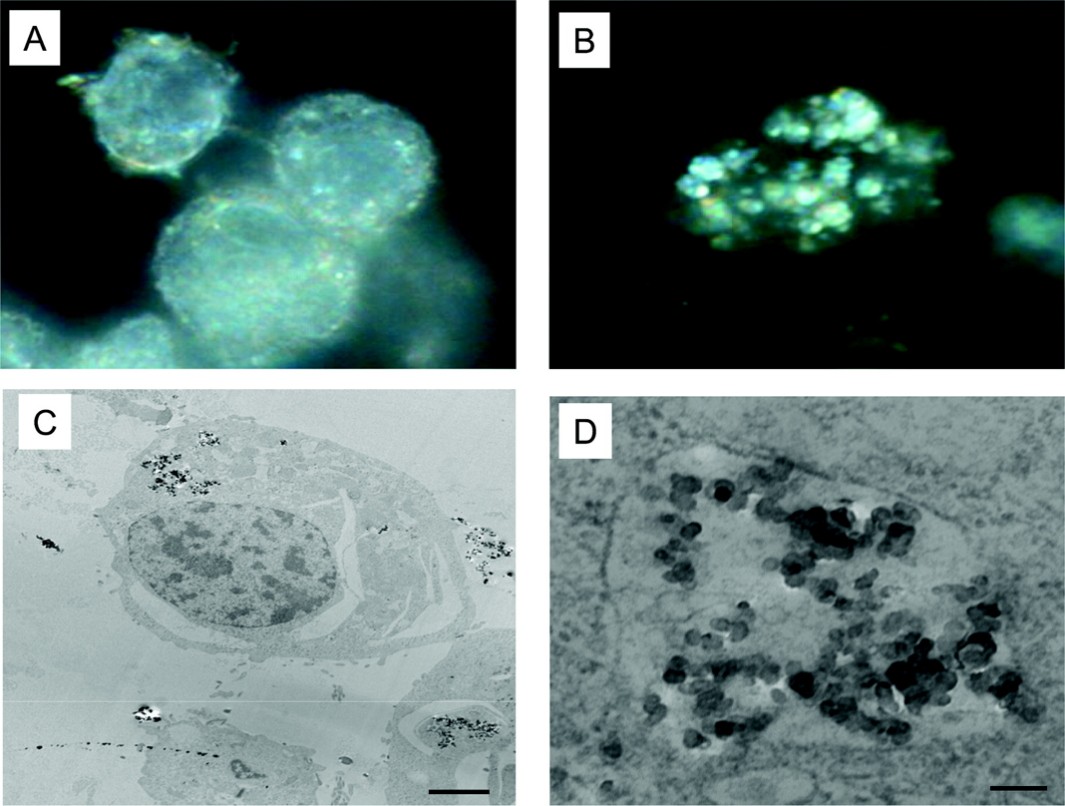
Examination of AgNP uptake by macrophages. (A) Control cells and (B) cells treated with 25 µg/mL of 30 nm AgNPs. (C) TEM micrograph of cells treated with 25 µg/mL of 55 nm AgNPs after 24 h of incubation and (D) magnified micrograph of the cell in (C) showing the accumulated AgNPs. Reprinted with permission from [102], Copyright 2008 American Chemical Society.

The human immune system adapts over time to recognize specific pathogens more efficiently. By doing so it provides an improved and lasting response to secondary encounters with the same specific pathogen (adaptive immunity) [15]. Conversely, the innate immune system is activated when an immediate response to pathogens is necessary and, in general, it is not a long-lasting protective immunity (immunologic memory). Monocytes and macrophages are the most common phagocytic cells in the body and represent the first innate line of defense, in addition to being responsible for the removal of particles [126]. Carlson et al. and Castillo et al. verified the interaction between AgNPs and macrophages and they saw that these NPs remained intact, with no evidence of AgNPs dissolution or cytotoxicity. Once inside the cells, and after 24 h of exposure, the nanoparticles remained at approximately the same size they were before incubation and uptake by the cells, as shown in Figure 7 [102], and they showed a potent ability to activate macrophages to produce ISGs and pro-inflammatory cytokines [115].

#### Toxicity of AgNPs

For a long time, silver was considered a safe antibacterial agent. The only reported side effect was argyria, which is a condition characterized by pigmentary changes secondary to exposure to silver salts which accumulate in the skin and mucous membranes. The toxicity of AgNPs is closely related to the release of Ag^+^ [57]. Studies with human mesenchymal stem cells (hMSCs) treated, under cell culture conditions, with different concentrations of Ag^+^ for 24 h indicate that a concentration of 2.5 ppm decreased cell viability by 60% whereas 5.0 ppm decreased cell viability by 100% in comparison to the control case. On the other hand, 30 ppm of AgNPs prepared immediately before the biological tests, in a system without oxygen and with a carrier gas to expel all dissolved oxygen (to prevent oxidation and release of silver ions) reported no reduction in cell viability compared to control [127].

Treatment of burns in rats with AgNPs were carried out both in vitro and in vivo. No significant differences in the levels of urea, creatinine, and aminotransferases and also in the hematological parameters were observed between the control and groups with burn wounds and between groups treated with AgNPs and groups with burn wounds. In addition, there were no significant differences in lipid peroxidation, carbonyl and reduced glutathione (GSH) protein levels between the groups [83,128].

Human exposure to silver occurs mainly through three different routes, viz. dermal, oral, and through inhalation. After exposure, AgNPs can potentially get accumulated within secondary organs, including the liver, spleen, and brain. Although a large amount of data is available on the applicability and toxicity of AgNPs, there are no standard procedures for preparing AgNPs or for assessing their toxicity [129]. Munger et al. conducted an oral in vivo exposure to commercial solutions of 10 and 32 ppm of AgNPs in healthy individuals (*n* = 60) who underwent metabolic and blood tests, urinalysis, sputum induction, and magnetic resonance imaging of the chest and abdomen. After oral exposure, the silver content in the serum and urine was analyzed and no clinically abnormal changes were noted in the lungs, heart, or abdominal organs. Also, no morphological changes were detected. In addition, no significant changes were observed regarding reactive pulmonary oxygen species or in the increase of pro-inflammatory cytokines [130].

The Australian health agency reports on the oral toxicity of AgNPs say that: “Studies report low toxicity in rats, mice and guinea pigs after ingestion (swallowing). In these studies with rodents, the degree of toxicity depended on the size of the particles and the dose administered.” [131].

The in vivo oral exposure to these commercial solutions of nanoscale silver particles does not lead to clinically important changes in metabolic, hematological, urinary, physical, or morphological findings. Further studies, however, are needed to observe the threshold toxicity in other human organs upon increasing dosage and time exposure to AgNPs [130].

The toxicity of AgNPs has been evaluated in several studies; however, only a few researchers considered the ion content (Ag^+^) of those nanoparticles. Toxicity assays could be performed by using oxidative stress and other reliable markers, such as 8-oxoguanine DNA glycosylase 1 (OGG1) and nuclear factor erythroid 2-related factor 2 (Nrf2) [132] or 3-(4,5-dimethylthiazol-2-yl)-2,5-diphenyltetrazolium bromide (MTT) to measure the number of viable cells, where viability is quantified by the reduction of MTT (tetrazolium) (yellow color), by the action of mitochondrial reductase, formazan (purple color) and this reaction only occurs in living cells [133–134], among other techniques. Therefore, the correct separation of the species is necessary to observe whether the toxicity is caused by the Ag^+^ ions, AgNPs, or by both. More details regarding the toxicity of the AgNPs and methods to separate the species are described in the section “Separation of AgNPs from Ag^+^” [135].

### Synthesis of silver nanoparticles

There are several synthesis methods that allow for the obtainment of different types of AgNPs with various shapes, sizes, and stability conditions [136]. Basic principles of some classic synthesis techniques are detailed in the following sections.

#### Citrate ion reduction

**Figure 8:**
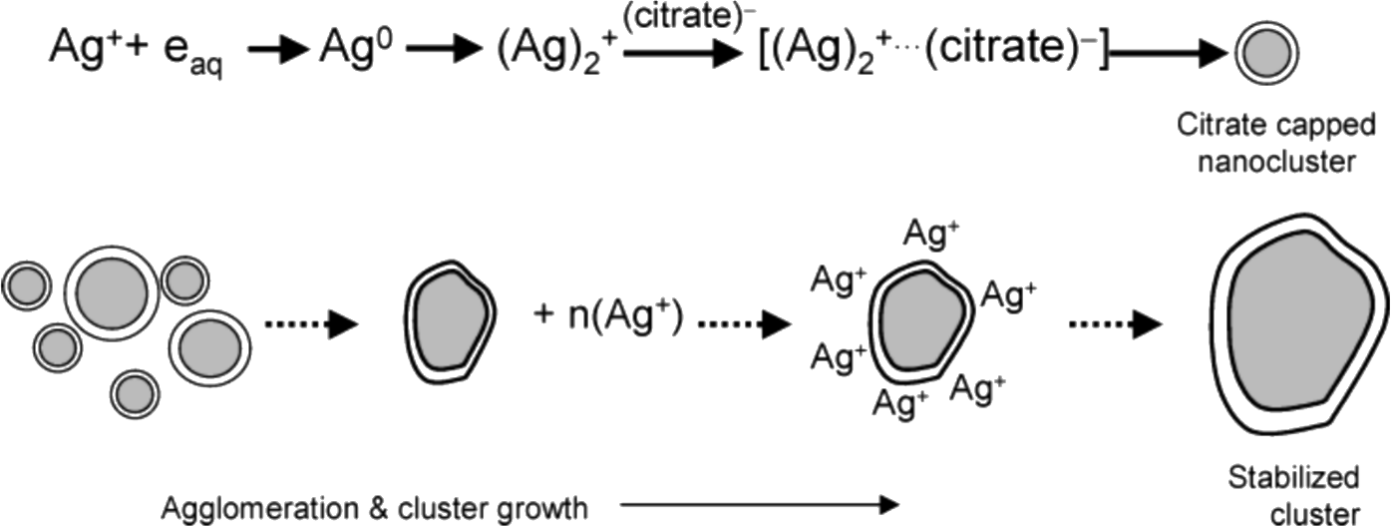
Primary and secondary growth steps in the formation of AgNPs through the citrate ion reduction technique. Reprinted with permission from [137], Copyright 2004 American Chemical Society.

Turkevich et al. reported the synthesis of gold nanoparticles in solution using trisodium citrate to reduce AuCl_4_^−^. Since then, this methodology is popularly known as the Turkevich method and it has been extended to other metals such as silver [137–138]. Following the same principle, this method is used to reduce Ag^+^. The stages of nucleation and growth are depicted in Figure 8.

**Figure 9:**
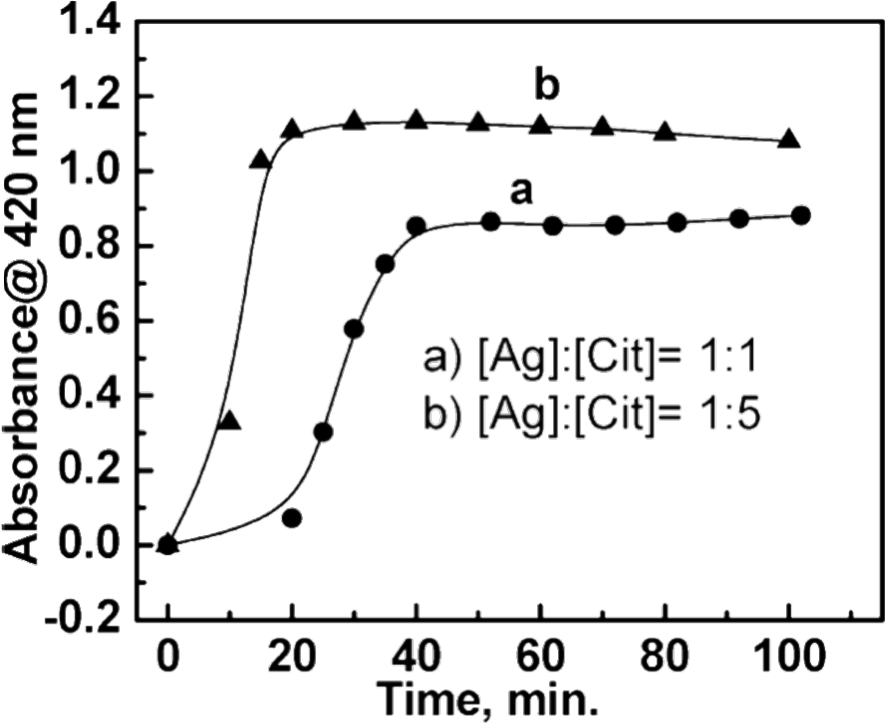
Graph depicting the variation in the reduction reaction time with respect to the [Ag^+^]/[citrate] ratio in the presence of 1 mM of AgNO_3_ and either (a) 1 mM or (b) 5 mM of sodium citrate as the reducer. Reprinted with permission from [137], Copyright 2004 American Chemical Society.

By modifying the temperature and the proportion of citrate concentration in relation to Ag^+^, Pillai and Kamat investigated the action of citrate in controlling the size and shape of AgNPs. By increasing the relative concentration of sodium citrate with respect to the silver cation (i.e., [Ag^+^]/[citrate] = 1:5), the time required for the formation of AgNPs was reduced from 40 to 20 min when compared to a 1:1 ratio of [Ag^+^]/[citrate]. However, a fraction of Ag^+^ was not reduced according to Figure 9 [137].

#### Synthesis with polyols and polyvinylpyrrolidone

The polyol synthesis was presented by Fievét et al. (1989) as a versatile synthesis route for various metallic and bimetallic nanoparticles, viz*.* Ag, Au, Cu, Co, Ni, Pd, Pt, CoNi, and FeNi with the possibility to obtain different shapes and sizes [139–140]. In this process, a suitable solid inorganic compound is suspended in a liquid polyol and the suspension is stirred and heated to a certain temperature, which can approach the boiling point of the polyol [140]. The versatility that the polyols offer in the obtainment of AgNPs with different shapes and sizes makes this method interesting for studying other forms of AgNPs besides the spherical ones, which are the most common [139]. Typically, the metal ion reduction reaction can be explained by the oxidation of two molecules of acetaldehyde produced by the dehydration of ethylene glycol (EG), as described in Equation 3 [104]:

[3]
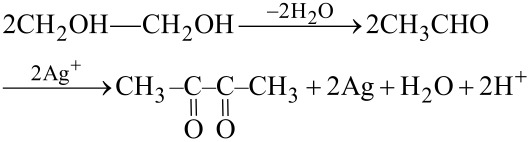


**Figure 10:**
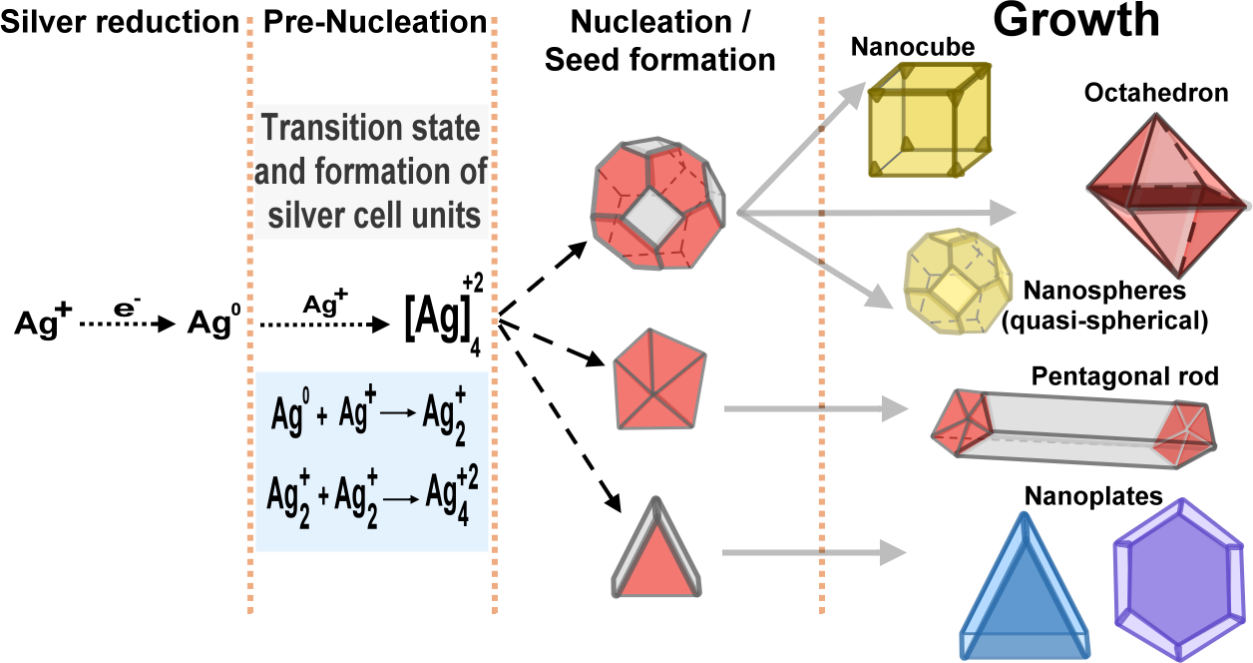
Schematic illustrating the reduction of silver ions, pre-nucleation, nucleation and growth steps in the formation of AgNPs with different shapes (e.g., nanocubes, octahedron, nanospheres, nanorods, and nanoplates) via the polyol synthesis process.

In summary, the polyol synthesis method involves the reduction of a metal salt in the presence of a boiling solvent at elevated temperatures (>160 °C). In order to protect the nucleated particles and avoid agglomeration, the most commonly used adjuvant is PVP [139]. One of the advantages of this method is that ethylene glycol, besides serving as a solvent, is also a reducing agent. Furthermore, as the reaction is dependent on the temperature it allows for an easy control of the nucleation and growth of AgNPs, as shown in Figure 10 [141].

Upon starting the reaction in an EG solution heated at 160 °C, the monocrystalline silver seeds are formed through homogeneous nucleation. The as-formed AgNPs seeds are dissolved by HNO_3_ present at a fairly high concentration during the initial stages of the reaction, according to Equation 4. After HNO_3_ is gradually consumed, a second round of nucleation is triggered, which favors the growth of AgNPs with various morphologies, such as nanocubes, nanowires, and nanospheres, as shown in Table 3 [139–140,142].

[4] $$4{\text{HNO}}_{3}+3\text{Ag}\underset{}{\to }3{\text{AgNO}}_{3}+\text{NO}+2{\text{H}}_{2}\text{O}$$

**Table 3:** Examples of the polyol process to obtain AgNPs with various morphologies.

[Ag^+^]	EG	protector	[Ag^+^]:[Protector]	reaction details (temperature/acid content/time)		AgNP morphology	ref.

94 mM	141 mM	PVP	1:1.5	EG/140 °C/0.25 mM HNO_3_	15 h	nanocubes 30 nm	[142]
94 mM	147 mM	PVP	1:1.5	EG/140 °C/0.025 mM HCl	30 min	nanowires ≈30 nm (d) ≈100 nm (c)	[143]
125 mM	188 mM	PVP	1:1.5	EG/160 °C	30 min	nanocubes ≈80 nm	[139]
250 mM	375 mM	PVP	1:1.5	EG/160 °C	45 min	nanocubes ≈175 nm	[139]
0.5 mM	0.0625 mM	PVP	1:0.0625	PEG/260 °C	24 h	nanospheres ≈54 nm	[144]
0.25 M	0.027 M	PVP	1:0.108	EG/120 °C	30 min	nanospheres ≈27 nm	[145]

### Minimum requirements for AgNP quality control

The physical and chemical properties of nanoparticles are important for the study of their behavior, biodistribution, safety, and effectiveness. Therefore, the characterization of AgNPs is important to assess the functional aspects of these NPs and it is essential for the establishment of regulatory guidelines to ensure safety in their use. The characterization of AgNPs is performed using a variety of analytical techniques, including ZP, UV–vis spectroscopy, inductively coupled plasma optical emission spectrometry (ICP-OES), dynamic light scattering (DLS), among other techniques [33]. The basic principles of the techniques mentioned here are detailed in the following sections.

Each AgNPs is unique, depending on its size, state of aggregation, physicochemical or biological synthesis methods used, chemical nature of the coating, surface charge, and free Ag^+^ content. The quality control of AgNPs is necessary to guarantee their suitability for the intended use, reproducibility, efficacy, and low toxicity [146]. Another important point is to confirm the importance to use the concentration in terms of number of particles (i.e., picomolar, pM/mL, or nanomolar, nM/mL) instead of the mass concentration (mg/L). The details of this item will be explained in the subsection “Separation of AgNPs from Ag^+^”.

#### Identification and quantification of AgNPs and Ag^+^

The ICP-OES technique is one of the most popular techniques used to identify metal ions and it allows us to detect AgNPs and Ag^+^ with high precision and sensitivity. This technique atomizes both Ag^+^ and AgNPs in the plasma and does not differentiate ionic silver (Ag^+^) from AgNPs [147].

**Separation of AgNPs from Ag^+^:** The cloud point extraction (CPE) technique was initiated by Goto et al. and it is based on the solubilization capacity and on the cloud point of nonionic surfactants [148]. At the cloud point, the instability observed in the micelles is promoted by the dehydration of the hydrophilic groups with consequent formation of giant micelles (cages) providing reduced water solubility (which defines the cloud point transition). It is exactly at this point that the AgNPs get surrounded by these non-ionic micelles and separated from the ionic silver (hydrophilic character) [149]. The protocol for this procedure is in the Supporting Information File 1, section “Procedure for separating AgNPs from Ag^+^.

**Quantification of AgNPs, Ag^+^, and total Ag species:** After centrifuging the CPE stage, the AgNPs decanted at the bottom whereas Ag^+^ remained in the supernatant, as shown in Supporting Information File 1, Figure S1. Thus, when analyzing the supernatant via ICP-OES, we only have the Ag^+^ content, which is a fundamental control parameter [149]. The concentration of AgNPs is given by the equation below:


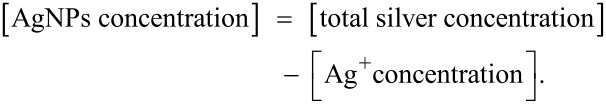


The mass content (mg/L) of AgNPs must be converted to number of particles/mL, which is related to its shape and size. To clarify, let's consider the following example wherein we have a solution of AgNPs with a nanosphere-like morphology with an average particle size of 20 nm. In the ICP-OES results for AgNPs, the content found was 30 mg/L (mass concentration). The calculation for this example is described in Supporting Information File 1, section “Calculating particles/mL for spherical AgNPs”, and the result found was 6.83 × 10^11^ particles/mL or 1.13 pM/mL. The same concentration of AgNPs found in the previous example would have had a totally different particle concentration if the average particle size was 5 nm. In this case, the results would be 4.37 × 10^13^ particles/mL and 72.58 pM/mL. For this reason, analytical correlation is essential for an accurate evaluation of the results. Table 4 and Table 5 show the correlation between mass concentration and particle concentration as a function of the size of the spherical AgNPs. In Supporting Information File 2 we provided a spreadsheet that allows for the immediate conversion of the mass concentration (ppm) of AgNPs to the concentration in particles/mL or pM/mL with respect to the average size and for different shapes of nanoparticles.

**Table 4:** Conversion table from mass concentration (mg/L) to particle concentration in pM/mL depending on the size of the spherical AgNPs.

mass concentration (mg/L)	10	20	30	40	50

diameter of spherical AgNPs (nm)	concentration (pM/mL)				

2.5	193.6	387.1	580.7	774.2	967.8
5.0	24.19	48.39	72.58	96.78	121.0
7.5	7.169	14.34	21.51	28.68	35.84
10	3,024	6.049	9,073	12.10	15.12
20	0.378	0.756	1,134	1.512	1,890
30	0.112	0.224	0.336	0.448	0.560
40	0.047	0.095	0.142	0.189	0.236
50	0.024	0.048	0.073	0.097	0.121
60	0.014	0.028	0.042	0.056	0.070
70	0.009	0.018	0.026	0.035	0.044
80	0.006	0.012	0.018	0.024	0.030

When multiplying the picomolar concentration by 10^−12^ and by the Avogadro constant (*N*_A_ = 6.02214076 × 10^23^ mol^−1^) [150], we have the number of particles/mL, as shown in Table 5. Morones et al. made use of the concentration of AgNPs (particles/mL) and correlated this with the concentration of colony forming units per mL (CFU/mL). In this example, the concentration values of AgNPs were ≈9.8 × 10^10^ particles/mL and the bacterial culture used in the study showed an optical density (OD) of 0.5, which corresponds to ≈5 × 10^7^ CFU/mL. The ratio between the number of AgNPs and the CFU was ≈2000 [8].

**Table 5:** Conversion table from mass concentration (mg/L) to concentration in number of particles/mL depending on the size of the spherical AgNPs.

mass concentration (ppm)	10	20	30	40	50

size of spherical AgNPs (nm)	concentration in number of particles/mL				

2.5	1.17 × 10^14^	2.33 × 10^14^	3.50 × 10^14^	4.66 × 10^14^	5.83 × 10^14^
5.0	1.46 × 10^13^	2.91 × 10^13^	4.37 × 10^13^	5.83 × 10^13^	7.29 × 10^13^
7.5	4.32 × 10^12^	8.63 × 10^12^	1.30 × 10^13^	1.73 × 10^13^	2.16 × 10^13^
10	1.82 × 10^12^	3.64 × 10^12^	5.46 × 10^12^	7.29 × 10^12^	9.11 × 10^12^
20	2.28 × 10^12^	4.55 × 10^11^	6.83 × 10^12^	9.11 × 10^11^	1.14 × 10^12^
30	6.75 × 10^10^	1.35 × 10^11^	2.02 × 10^11^	2.70 × 10^10^	3.37 × 10^11^
40	2.85 × 10^10^	5.69 × 10^10^	8.54 × 10^10^	1.14 × 10^11^	1.42 × 10^11^
50	1.46 × 10^10^	2.91 × 10^10^	4.37 × 10^10^	5.83 × 10^10^	7.29 × 10^10^
60	8.43 × 10^9^	1.69 × 10^10^	2.53 × 10^10^	3.37 × 10^10^	4.22 × 10^10^
70	5.31 × 10^9^	1.06 × 10^10^	1.59 × 10^10^	2.12 × 10^10^	2.65 × 10^10^
80	3.56 × 10^9^	7.11 × 10^9^	1.07 × 10^10^	1.42 × 10^10^	1.78 × 10^10^

**UV–vis–NIR absorption spectroscopy:** Metallic nanoparticles are known to emit characteristic colors in the visible region of the electromagnetic spectrum due to a phenomenon known as surface plasmon resonance. The color of a colloidal nanoparticle solution is mainly dependent on the size and shape of the nanoparticles [63]. The UV–vis absorption spectrum is generally recorded between 210–1100 nm for AgNPs with various morphologies. By using the UV–vis absorption spectrum the physical and chemical properties of the nanoparticles can be correlated. The optical properties of the AgNPs tend to change when the particles aggregate and the conducting electrons closer to each particle surface are relocated and shared between neighboring particles. This causes a change in surface plasmon resonance which can be observed from the absorption spectrum. It is also conceivable to evaluate a possible dissolution of AgNPs, due to the fact that the silver ion does not absorb in the visible region [55,63].

Zhang et al. carried out a systematic study of the production of spherical AgNPs with PVP and citrate. The reactions produced spherical AgNPs which exhibited an acute plasmon resonance peak at ≈400 nm in the UV–vis absorption spectrum. However, by modifying the reaction medium with peroxide, citrate, and PVP they noticed the formation of AgNPs in the form of nanoplates, which had an absorption peak at ≈560 nm [151].

**Figure 11:**
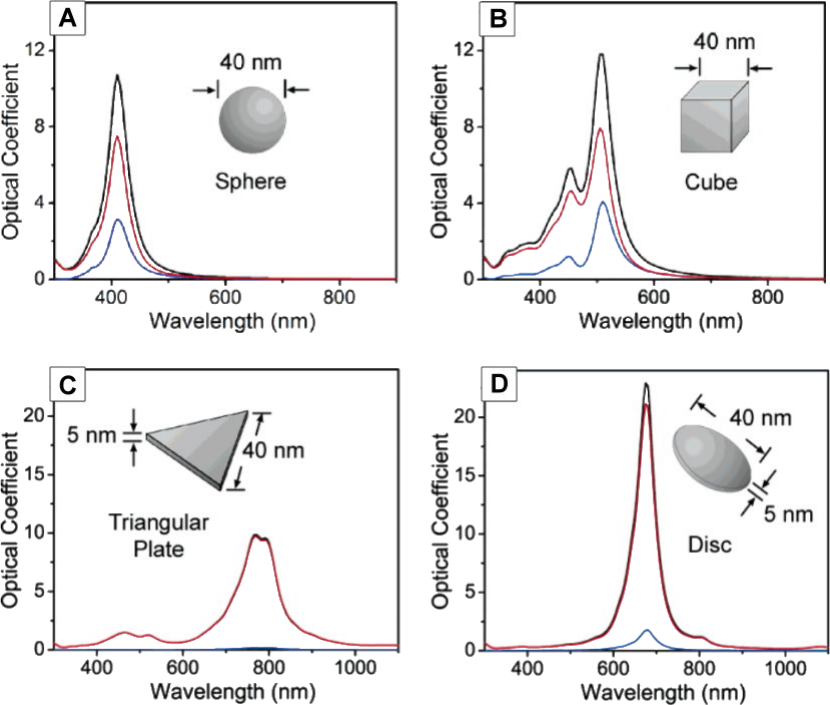
Calculated UV–vis extinction (black), absorption (red), and scattering (blue) spectra of silver nanostructures, illustrating the effect of the shape of a nanostructure on its spectral characteristics. An isotropic sphere (A) exhibits spectra with a single resonance peak. The spectra of an anisotropic cube (B), a triangular plate (C), and a circular disc (D) are also shown. Reprinted with permission from [63], Copyright 2006 American Chemical Society.

Gustav Mie was the first to rigorously explain the colors exhibited by metal colloids by using Maxwell's equations. Following the discrete dipole approximation (DDA), it is possible to calculate the dipole polarized by the incident light and all the other dipoles in the nanoparticle matrix, in addition to predicting the behavior of their respective spectra. Figure 11 shows the extinction, absorption, and scattering spectra by UV–vis (extinction spectrum = absorption + scattering) for AgNPs in water, calculated by using the Mie scattering theory [63].

**Figure 12:**
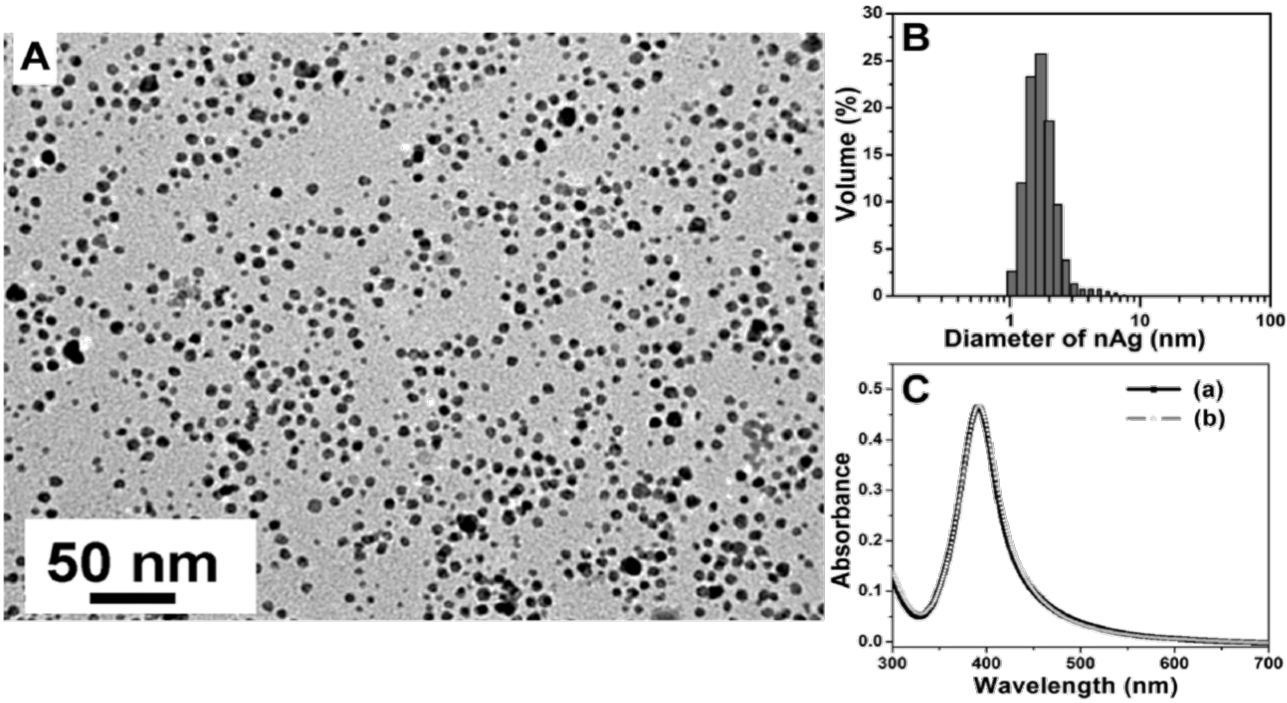
Experiment investigating the stability of AgNPs. (A) TEM micrograph after sample drying. (B) In situ size distribution by dynamic light scattering. (C) UV–vis absorption spectra of AgNPs suspended in DI water: (a) freshly synthesized or (b) stored at 4 °C for five months. The peak absorbance at 390 nm was found to remain unchanged indicating size and shape stability. Reprinted with permission from [55], Copyright 2010 American Chemical Society.

UV–vis spectroscopy is a simple and safe method to monitor the stability of AgNPs. When particles aggregate, dissociate, or change shape, the original absorption peak exhibits a change in intensity or position, which clearly indicates a variation. Experimentally, the stability of AgNPs was studied as depicted in Figure 12. The UV–vis absorption spectrum of the as-prepared sample was almost comparable to the spectrum obtained for a five-month-old AgNPs sample stored at 4 °C leading to an overlap, which indicates size and shape stability of the nanoparticles [48].

**Dynamic light scattering:** Historically, the first approaches to describe interactions between light and nanostructures were published in the early 20th century. Currently, the most popular theory is the one published by Gustav Mie in 1908. Briefly, this theory describes that the intensity of the scattered light of an incident laser radiation (λ = 633 nm) is dependent on the size of the particles. Light scattering theories are easily applicable to spherical and non-interacting particles whereas new models have been developed for other types of more complex structures [66,152]. When the particle sizes are 1/10 of the wavelength of the incident light (i.e., particles smaller than 63 nm in size for a laser wavelength of 633 nm), the scattered light carries the same energy (elastic dispersion) as the incident light and does not depend on the angle (Rayleigh scattering). However, when the particle size is greater than 63 nm, then the Rayleigh dispersion is no longer valid and it is replaced by the Mie anisotropic dispersion theory, in which the scattered light energy is different from the incident light energy (inelastic dispersion) and is dependent on the angle [153].

**Figure 13:**
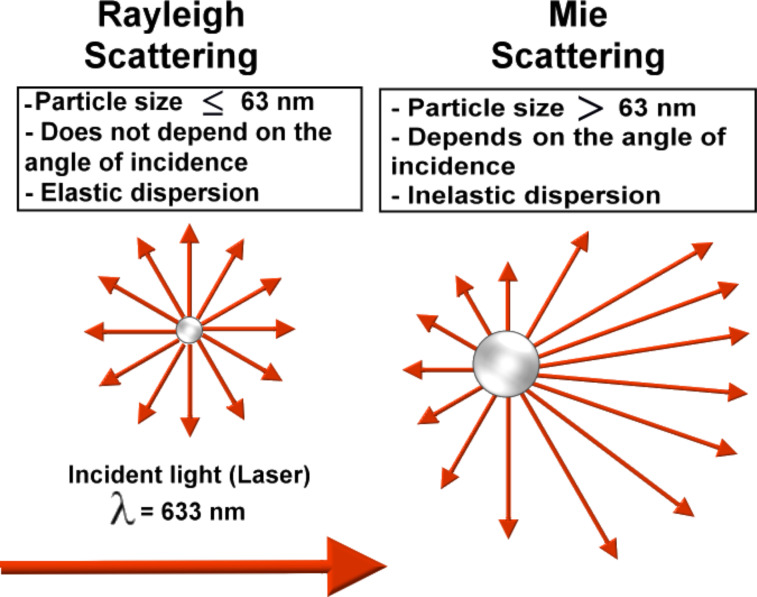
Schematic representing the differences between Rayleigh (left) and Mie (right) light scattering theories.

Data referring to the dispersing medium (e.g., refractive index and viscosity) and the dispersed nanoparticle (e.g., refractive index and absorption), when it comes to nanoparticles ≤63 nm, are not required by the Rayleigh model. In contrast, for particles >63 nm this information is essential to obtain the correct result [154]. Figure 13 shows the difference between the light scattering theories of Rayleigh and Mie.

The DLS technique provides important information for the evaluation of mono- and polydispersed solutions (i.e., the polydispersion index, PDI) which is basically a representation of the size distribution of the population in a given sample. The numerical value of the PDI ranges from 0 (for a perfectly uniform sample in terms of particle sizes) to 1.0 (for a highly polydispersed sample with a population with several particle sizes). Values less than or equal to 0.3 are considered acceptable and indicate a homogeneous population whereas values greater than 0.3–0.6 indicate a population with an average polydispersity. Values above 0.7 indicate a very polydispersed population [155]. In a complementary way, the size, distribution, shape heterogeneity, morphology, dispersion, and aggregation can be directly evaluated via TEM in which the high spatial resolution facilitates the investigation of the electronic structure and chemical composition [156]. However, the disadvantages other than the expensive instrument cost include the requirement of high voltage, high vacuum and a tricky sample preparation protocol, which is extremely important to obtain high-quality images [33,156].

**Zeta potential:** The zeta potential, also called the electrokinetic potential, is the potential in the sliding plane of a particle that moves under an electric field. The ZP reflects the potential difference between the double electric layer of electrophoretically mobile particles and the dispersant layer around them in the sliding plane [153]. AgNPs with ZP between −30 and −125 mV are considered to be strongly anionic and stable. Another important point is that with the increase in ionic strength the diffuse layer becomes more compact while the ZP decreases, and vice versa [153].

**X-ray diffraction:** The principle of the X-ray diffraction (XRD) technique is based on Bragg's law. The crystallographic planes of a crystal act as mirrors so that the X-ray beams are reflected in a reflected wavefront forming a second angle, θ [33]. The Bragg's Law relates the incident wavelength, λ, to the interplanar spacing, *d*, between the planes. When the specified conditions are met, we have: *n*λ = 2Δ = 2*d*sin θ [157]. The crystalline structure, size, and shape of the unit cell of a silver nanoparticle can be determined using XRD. The Bragg reflexes of the face-centered cubic structure (FCC) of AgNPs are generally observed at 2θ (Bragg angle) = 38.00°, 44.16°, 64.40°, and 77.33° and correspond to (111), (200), (220), and (311) planes, respectively [158]. Powder XRD is effective in studying the crystalline structure of heterogeneous nanoparticles and nanostructures [159]. As the 2θ angle increases, the phase difference between two progressive waves also increases and the scattering intensity decreases [159]. Nanoparticles with sizes smaller than 100 nm can have their crystallite size estimated by the Scherrer formula, according to Equation 5:

[5] $$D_{\text{XDR}}=\frac{K\lambda }{\beta \cos\theta },$$

where, *K* is the Scherrer constant (*K* = 0.89 rad), λ (nm) is the X-ray wavelength, β (rad) is the peak width at half the maximum intensity, and θ (rad) is the Bragg angle [160].

## Conclusion

The remarkable biological properties of AgNPs as antiviral and antibacterial agents draw the attention to the development of new products in the healthcare sector. The mechanism of action of AgNPs against bacteria and viruses has been analyzed and that knowledge will help in a better understanding of the use of silver as an antimicrobial agent with high efficiency and low toxicity. Studies indicate safe use in vivo and in the human body. Several studies also show that the AgNPs bactericidal action mechanism is related to the {111} facets and, therefore, shapes that have a greater number of {111} facets, such as triangular nanoplates, have higher antibacterial activity when compared to spherical nanoparticles.

In the analyses of the studies involving AgNPs at the chemical, pharmacological, biological, and toxicological levels, it was observed that AgNPs present behaviors and mechanisms distinct from the silver ion (Ag^+^). From the point of view of quality control, for a sensible assessment and to include all studies involving AgNPs, the identification and quantification of these species should be considered. This review addresses the methods of identification and quantification of these species, which should be required in any scientific work involving silver nanoparticles.

Regarding the physical and chemical properties of AgNPs, special attention was given to oxidative dissolution and photoaging, where we explained the importance of the storage temperature to be between 2 and 8 °C and the need to always keep the samples protected from light (solar and artificial). In addition, we showed that pH values higher than 9.0 and the absence of dissolved oxygen during synthesis are essential intrinsic factors to promote good stability of AgNPs.

Therefore, the characterization of AgNPs is important to assess safety, efficacy, and toxicity. Quality control plays an essential role in ensuring suitability for the intended use, reproducibility, efficacy, and toxicity. In this review, some of the main characterization techniques are mentioned for a better understanding of the methods used. Another considerable point is to confirm the importance to use the concentration in terms of number of particles/mL, picomolar (pM/mL), or nanomolar (nM/mL) instead of using the mass concentration (mg/L). This is due to the fact that, when working with nanoparticles, the unit that standardizes a clinical study becomes the quantity of particles and not the mass anymore. Conversion tables for pM/mL and number of particles/mL for particles with spherical (or quasi-spherical) shapes ranging from 2.5 to 80 nm with mass concentration ranging from 10 to 50 mg/L were presented.

Finally, considering that AgNPs can be used in biological applications it is necessary to assess their benefits and risks. In terms of toxicity and cytoxicity, again, it is necessary to identify and differentiate the species of AgNPs and Ag^+^ in order to realistically understand how they influence pharmacodynamics and their interactions with cells and living organisms. Multidisciplinary teams are essential to pave the way for the future use of AgNPs in living beings.

## Supporting Information

File 1 Method to separate silver species and to calculate particle concentration of spherical AgNPs.

File 2 Conversion from mass concentration (ppm) to particle concentration of AgNPs with different shapes.
