# Supplementary material for: A review on nanostructured silver as a basic ingredient in medicine: physicochemical parameters and characterization
Source: Beilstein J Nanotechnol. 2021 May 14;12:440–61. doi: 10.3762/bjnano.12.36 (PMC8144915; doi:10.3762/bjnano.12.36)
Supplement: File 1 — Method to separate silver species and to calculate particle concentration of spherical AgNPs. [file Beilstein_J_Nanotechnol-12-440-s001.pdf]

BEILSTEIN JOURNAL OF NANOTECHNOLOGY

# Supporting Information

for

# A review on nanostructured silver as a basic ingredient in medicine: physicochemical parameters and characterization

Gabriel M. Misirli, Kishore Sridharan and Shirley M. P. Abrantes

*Beilstein J. Nanotechnol.* **2021,** *12,* 440–461. doi:10.3762/bjnano.12.36

## Method to separate silver species and to calculate particle concentration of spherical AgNPs

License and Terms: This is a supporting information file under the terms of the Creative Commons Attribution License (https://creativecommons.org/licenses/by/4.0). Please note that the reuse, redistribution and reproduction in particular requires that the author(s) and source are credited and that individual graphics may be subject to special legal provisions.
The license is subject to the *Beilstein Journal of Nanotechnology* terms and conditions: (https://www.beilstein-journals.org/bjnano/terms)

# Procedure for separating AgNPs from $Ag^+$

This procedure follows the following steps. [1,2]:

1. Nonionic surfactant is added to the sample solution with a final concentration higher than its critical micellar concentration (CMC). This is the minimum concentration necessary to start the formation of micelles of a respective surfactant. Table S1 shows the technical information for Triton X-100, which is a typical nonionic surfactant.

2. The pH is adjusted with $HNO_3$ to 3.0 and sodium thiosulfate is added to decrease the zeta potential and to create electrostatic instability in the medium containing the AgNPs to facilitate separation.

3. The temperature is increased up to the cloud point of the surfactant.

4. The micellar solution is centrifuged while still heated, with subsequent separation in two phases. AgNPs are precipitated at the bottom and the silver ion remains in the supernatant.

**Table S1:** Information and properties of the Triton X-100 (Sigma-Aldrich) used in the experiment

| | |
|---|---|
| CAS Number: | 9002-93-1 |
| Chemical name: | *t*-octylphenoxypolyethoxyethanol |
| Molecular weight: | 625 g/mol |
| CMC: | 0.2–0.9 mM (20–25 °C) |
| Mist Point: | 65 °C |

In practical terms, the extraction of AgNPs can be carried out in a polypropylene tube (type “Falcon®”) in which 9.5 mL of the sample is added. In addition, 400 μL of Triton X-100 5% (w/v), 100 μL of 1M $Na_2S_2O_3$ (sodium thiosulfate), and approx. 10 μL of 1M

$HNO_3$ are also added to the samples with a maximum pH of 10 such that the pH lowers to approx. 3.0. The samples are heated in a water bath until the cloud point reaches 65 °C. The sample is then centrifuged for 30 min. at 3000 rpm, as shown in Figure S1.

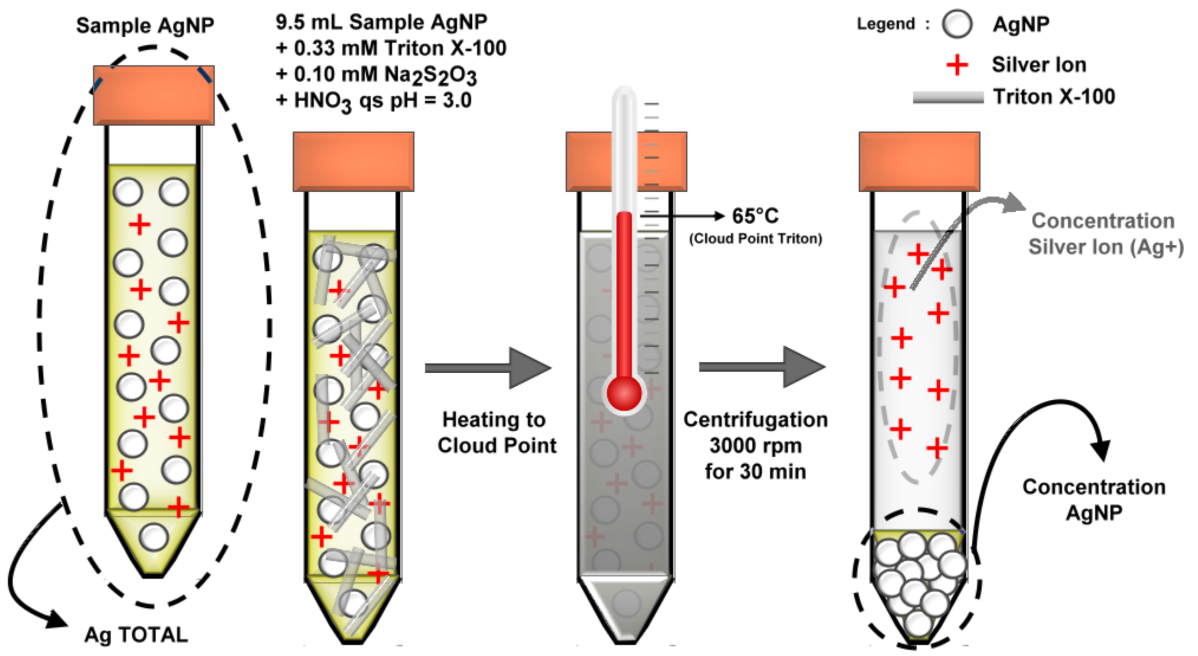


**Figure S1:** Procedure for separating AgNPs using the Cloud Point Extraction (CPE) technique.

# Calculating particles/mL for spherical AgNPs

Let's consider the example below:

Shape: Spherical

Size: 20 nm

Concentration: 30 mg/L (mass concentration).

Volume of a sphere:

$V = \frac{4\pi}{3} r^3$ , by replacing $r = \frac{d}{2}$, we have: $V = \frac{4\pi}{3} \frac{d^3}{2^3}$ (S1)

Where *d* = diameter of the sphere (cm), *r* = radius of the sphere:

$V = \frac{m}{\rho}$ (S2)

where ρ = density (g/cm³), *m* = particle mass (g). Replacing in Equation S1, we have

$\frac{m}{\rho} = \frac{4\pi}{3}\frac{d^3}{8}$ and $d^3 = \frac{6m}{\pi\rho}$

$$m = \frac{d^3 \times \pi \times \rho}{6} \qquad \text{(S3)}$$

The Equation S3 is the formula for determining the mass of one spherical Ag nanoparticle, where ρ = density of silver (10.49 g/cm³) [3], π = 3.14159 [4], *m* = particle mass (g), and *d* = particle diameter (cm).

By applying Equation S3 to our example, we have the mass of each particle:

$$m = \frac{(20 \times 10^{-7})^3 \times 3.14159 \times 10.49}{6}$$

$m = 4.39 \times 10^{-17} g$ (mass of one particle)

As the mass concentration found was 30 mg/L (or 0.030 mg/mL), we have:

$$N° \, Particles = \frac{30 \times 10^{-6} \, g \, AgNP}{4.39 \times 10^{-17} g}$$

$N° \, Particles = 6.83 \times 10^{11}$ particles / mL

The conversion of the number of particles/mL to pM/mL (picomolar) is done according to the following relation:

$$AgNP \, Conc. (pM/mL) = \left(\frac{6.83 \times 10^{11} \, particles}{6.02214076 \times 10^{23}}\right) \times 10^{12}$$

$AgNP \, Conc. = 1.13 \, pM/mL$

# References


1. Liu, J. F.; Liu, R.; Yin, Y. G.; Jiang, G. Bin. *Chem. Commun.* **2009**, No. 12, 1514–1516. https://doi.org/10.1039/b821124h.
2. Liu, J. F.; Yu, S. J.; Yin, Y. G.; Chao, J. B. *TrAC - Trends in Analytical Chemistry*. March **2012**, pp 95–106. https://doi.org/10.1016/j.trac.2011.10.010.
3. *Metals Handbook Desk Edition*; Davis, J. R., Ed.; ASM International, 1998.

https://doi.org/10.31399/asm.hb.mhde2.9781627081993.

4. Bailey, D. H.; Borwein, J. M.; Borwein, P. B.; Plouffe, S. **1997**, *19* (1), 50–56.
